# Project, toolkit, and database of neuroinformatics ecosystem: A summary of previous studies on “Frontiers in Neuroinformatics”

**DOI:** 10.3389/fninf.2022.902452

**Published:** 2022-09-26

**Authors:** Xin Li, Huadong Liang

**Affiliations:** ^1^School of Information Science and Technology, University of Science and Technology of China, Hefei, China; ^2^AI Research Institute, iFLYTEK Co., LTD, Hefei, China

**Keywords:** data life cycle, project, multi-modal database, toolkit, knowledge base

## Abstract

In the field of neuroscience, the core of the cohort study project consists of collection, analysis, and sharing of multi-modal data. Recent years have witnessed a host of efficient and high-quality toolkits published and employed to improve the quality of multi-modal data in the cohort study. In turn, gleaning answers to relevant questions from such a conglomeration of studies is a time-consuming task for cohort researchers. As part of our efforts to tackle this problem, we propose a hierarchical neuroscience knowledge base that consists of projects/organizations, multi-modal databases, and toolkits, so as to facilitate researchers' answer searching process. We first classified studies conducted for the topic “Frontiers in Neuroinformatics” according to the multi-modal data life cycle, and from these studies, information objects as projects/organizations, multi-modal databases, and toolkits have been extracted. Then, we map these information objects into our proposed knowledge base framework. A Python-based query tool has also been developed in tandem for quicker access to the knowledge base, (accessible at https://github.com/Romantic-Pumpkin/PDT_fninf). Finally, based on the constructed knowledge base, we discussed some key research issues and underlying trends in different stages of the multi-modal data life cycle.

## Introduction

Most chronic diseases in epidemiology take time to form, and many risk factors for the disease may cause the occurrence of diseases in this process. A longitudinal cohort study is a common research method in epidemiology, which is an effective way to obtain pathogenic risk factors and evaluate intervention measures based on the correlation between “exposure” and “outcome” (Louis and Tampone, 2019). In recent years, some large-scale longitudinal cohort studies have been carried out and achieved good results, such as IMAGEN (Schumann et al., 2010), ABCD (Luciana et al., 2018), and UK-Biobank (Littlejohns et al., 2020).

**Figure 1:**
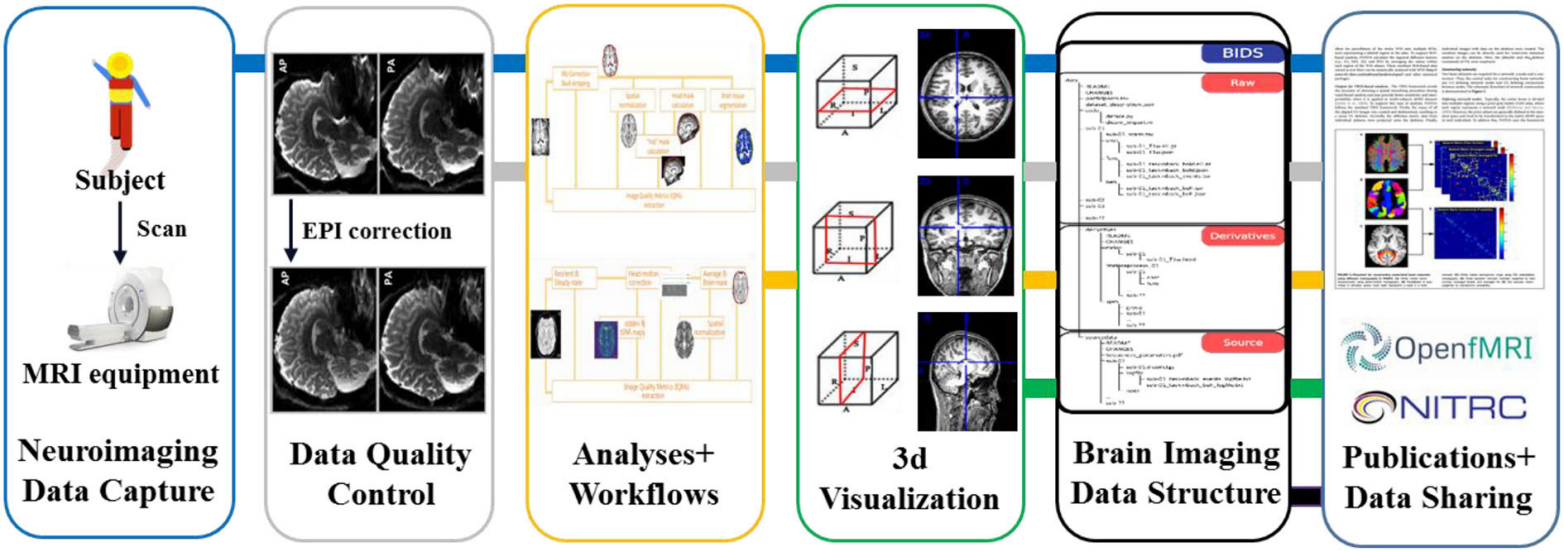
Data life cycle in neuroimaging research.

It can be seen that the core contents of the longitudinal cohort study are prospective in multi-modal data collection, multi-modal data analysis, and multi-modal data sharing. Take the neuroimaging data as an example, the whole data life cycle can be shown in Figure 1. However, in the process of traditional cohort construction, some major problems need to be solved urgently: (1) A variety of experimental data and metadata are collected and stored based on paper-based records; (2) The calculation efficiency of data quality control (QC) was low, and timely feedback on and corrections of the data quality are difficult to receive; (3) Data management standards are difficult to unify, and multi-modal data are difficult to integrate and share effectively. Therefore, in recent years many efficient and high-quality data information platforms, technologies, toolkits, and standards for cohort study construction have been published and applied in multiple cohort data research stages such as multi-modal data collection, data QC analysis, computational analysis modeling, and data sharing. Thus, researchers ultimately hope to improve the quality of multimodal data for cohort studies.

From the perspective of cohort construction researchers, how many related works have been published? What application effects have been achieved in the data life cycle of cohort construction? What other key issues need to be further resolved? With these questions, we tried to search the corresponding literature retrieval database, such as the Web of Science, to seek answers to these questions. However, most of the retrieved article topics focus on a single point of technology and method improvement. As a result, we did not find a complete matching study to answer the above questions. Therefore, to help researchers more efficiently retrieve and reference the existing technical and functional architecture solutions, we mainly make the following contributions in this study: (1) We proposed a hierarchical knowledge base framework consisting of projects, toolkits, and databases of the neuroinformatics ecosystem, and developed an open source knowledge base query tool, PDT_fninf, in order to help researchers quickly search the corresponding content from the knowledge base; (2) According to the content of the knowledge base, the main research progress, and trends in each stage of the data life cycle are analyzed and discussed, which provide some guidance for follow-up research.

The rest of this article will be organized as follows: (1) the “Methods” section is to describe the principle of categorizing articles topics in Frontiers in Neuroinformatics, and the construction process of the knowledge base, (2) sections 3~8 beginning with “Multi-modal” summarize and discuss the main contributions of existing studies and their underlying trends from different stages of the data life cycle, and (3) the section of “Conclusions” summarizes our main contributions and our future works.

## Methods

In order to complete the neuroinformatics ecosystem of cohort studies, we selected the Frontiers in Neuroinformatics journal as the input instances of the knowledge base in this study, which has published some works on existing neuroscience databases, and novel tools for data acquisition, analyses, visualization, and dissemination of nervous system data. Specifically, we first divided the Frontiers in Neuroinformatics journal articles into different topics according to the data life cycle of the cohort study. Then, the information objects in these articles are filled and associated with the knowledge base framework. Finally, we open source the corresponding knowledge base query tool based on the constructed knowledge base.

### Categorize articles by data life cycle

First, we searched all the articles published in this journal from 2007 to 2021 on the Web of Sience database and obtained a total of 723 articles. We imported them into “Endnote” software for grouped literature management. Then, two authors of this paper classified these articles in a double-blind mode, according to the initial categories associated with the cohort data life cycle, into six categories, “Multi-modal data collection”, “Multi-modal data quality control”, “Multi-modal data mining analysis”, “Multi-modal data visualization”, “Multi-modal data management” and “Multi-modal data sharing”. It is worth noting that the third person will introduce a centralized voting decision-making mechanism when some articles aren't uniformly classified or can't be classified. Finally, all articles are assigned a different category label as shown in Table 1. Meanwhile, the keywords list of these articles is also updated synchronously.

**Table 1:** The categories of all articles published in Frontiers in Neuroinformatics.

**Category**	**Keywords**	**Num**
Multi-modal data capture	Collect/acquisition/capture	6
Multi-modal data quality control	Quality control/assessment	5
Multi-modal data mining analysis	Analysis/pipeline/workflow/process/calculate	82
Multi-modal data visualization	Visualize/browse/view/3D/construct	48
Multi-modal data management	Manage/open science/xnat	47
Multi-modal data sharing	Sharing/atlas/dataset/database/	40
Others	BCI/review/survey/meta/reproduce/….	495

We mainly focus on the various stages of the data life cycle in the construction of the cohort data information platform. According to the article categories shown in Table 1, we summarized and discussed the main contributions of these 228 articles to the construction of the neuroinformatics ecosystem.

### A hierarchical knowledge base framework

Researchers mainly carry out different neuroscience research in the form of projects or working groups, such as the HCP project (Marcus et al., 2011) for mapping all the neural connections in the human brain and the ADNI Project for searching the biomarkers of Alzheimer's disease (Mueller et al., 2005; Jack et al., 2010). These projects collect multimodal data for solving different research problems. Meanwhile, at different stages of the data life cycle, a variety of information toolkits have been developed to support the implementation of these projects. Among them, the data types of multi-modal data mainly include clinical/behavioral data, neuroimaging data, electrophysiological data, and molecular data. The data life cycle stages mainly include data capture, data QC, data analysis, data visualization, data management, and data sharing.

**Figure 2:**
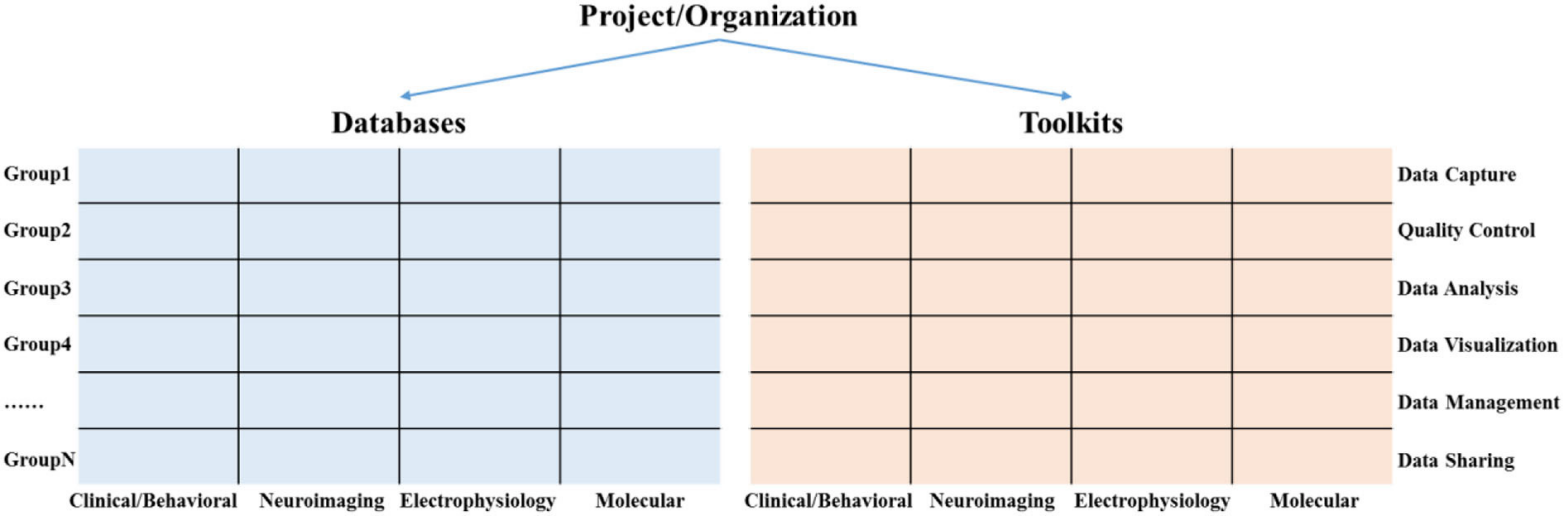
A hierarchical knowledge base framework consists of projects, databases, and toolkits.

In order to help researchers quickly sort out and trace the research progress of existing projects on multi-modal databases and information toolkits, we propose a hierarchical knowledge base framework that consists of projects, databases, and toolkits, as shown in Figure 2. The databases and toolkits are the main products of the project/organization. of these, the databases can be mapped into a matrix composed of multi-modal data types and different diagnostic groups. Similarly, the toolkits can be mapped into a matrix composed of multimodal data types and the data life cycle stage.

### Knowledge base filling

Information object recording: we used the “5W-4M-6P” information collection framework to summarize and sort information objects about the projects/organizations, databases, and toolkits, obtained from the 228 articles in the Frontiers in Neuroinformatics (Table 2).

**Table 2:** A summary and collation framework for projects, databases, and toolkits.

	**Column name**	**Meaning**
**5W**	Which	Annotation information objects belong to projects, databases, or toolkits.
	Who	Record the name of the information object in abbreviated (full name) format.
	What	Record the content or main function descriptions of the information object.
	Where	Record the source of the information object, including its project, published articles, and access address information.
	When	Record the generation time of the information object.
**4M**	Clinical/behavioral	Recordings about clinical information and reactions made in response to different stimulus of the subject.
	Neuroimaging	Produced brain images by noninvasive techniques (such as computed tomography and magnetic resonance imaging).
	Electrophysiology	Electrical signals associated with a physiological process (such as the function of a body or bodily part).
	Molecular	Data resources of, relating to, consisting of, or produced by molecules.
**6P**	Data Capture	Multi-modal data capture phase.
	Quality Control	Multi-modal data quality control phase.
	Data Analysis	Multi-modal data analysis stage.
	Data Visualization	Multi-modal data visualization stage.
	Data Management	Multi-modal data management phase.
	Data Sharing	Multi-modal data sharing phase.

**Figure 3:**
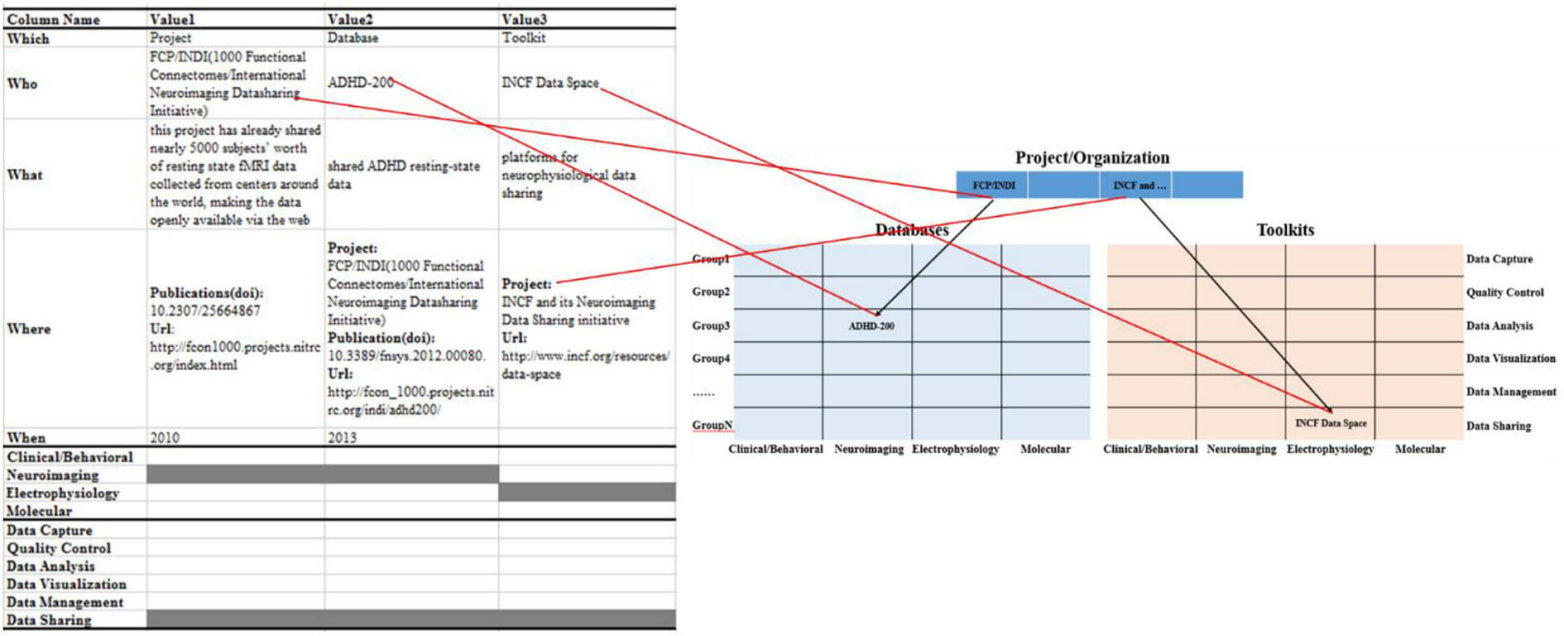
Map information objects to a hierarchical knowledge base framework.

Information object mapping: the summarized information objects were mapped into the proposed hierarchical knowledge base framework, as shown in Figure 3. Among them, we classify information objects into projects/organizations, multi-modal databases, or information toolkits modules based on the “Which” field. In the multi-modal databases and toolkits module, we associate the information object with a specific project/organization through the “Project” property in the “Where” field. Meanwhile, the data types and data life cycle phases involved in the information object are marked as gray in the “4M” and “6P” modules, respectively.

**Figure 4:**
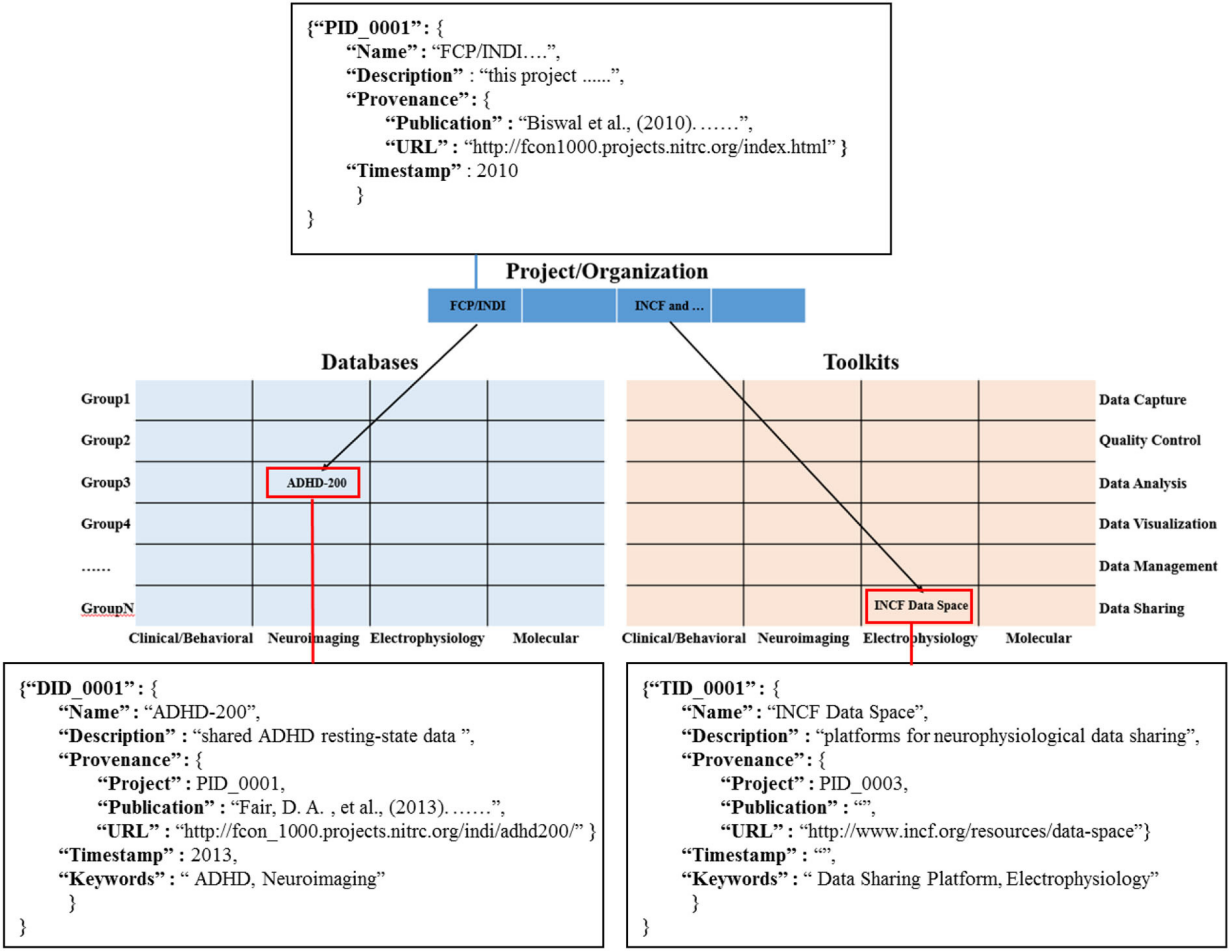
The information objects in knowledge base are coded and organized by dictionary.

Information object coding: in order to define the information object and its related connections, we construct the data dictionary for the information objects of different modules in the knowledge base (Figure 4). And, we use the primary foreign key to establish the connection between information objects in the projects/organization module (PID_0001) and the information objects in multi-modal databases (DID_0001) and the toolkits module (TID_0001). Among them, it is worth noting that in the multi-modal databases matrix and toolkits matrix, each cell is filled by a list element composed of similar information objects, such as both NDAR (National Database for Autism Research) and ABIDE (Autism Brain Image Data Exchange) provide a large amount of neuroimaging data for the study of the autism population. In addition, we use the “Keywords” field in the data dictionary to represent the cell position of the information objects (red rectangle) in the multi-modal databases matrix and toolkits matrix.

### Knowledge base query and statistics

**Figure 5:**
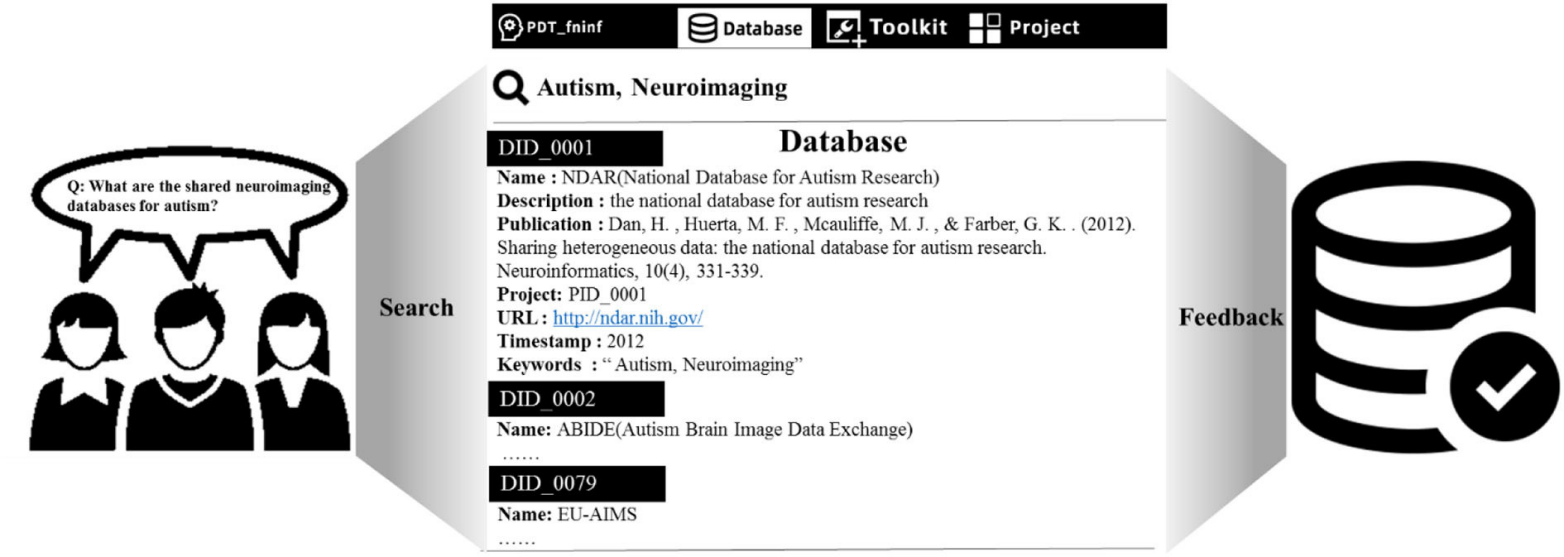
Query the neuroimaging database of autism from the knowledge base.

We developed PDT_fninf, a knowledge base query tool based on Python (https://github.com/Romantic-Pumpkin/PDT_fninf), which can help researchers access this knowledge base. As shown in Figure 5, researchers retrieved the answers to the question “What are the shared neuroimaging Databases for autism?” They can first click the “Database” button to enter the multi-modal database module. Then, they can give keywords in the search box, such as “Autism, Neuroimaging”. Finally, press Enter key to obtain the relevant information objects in the knowledge base, and more instructions can be found in Readme module in the above Github repository.

**Figure 6:**
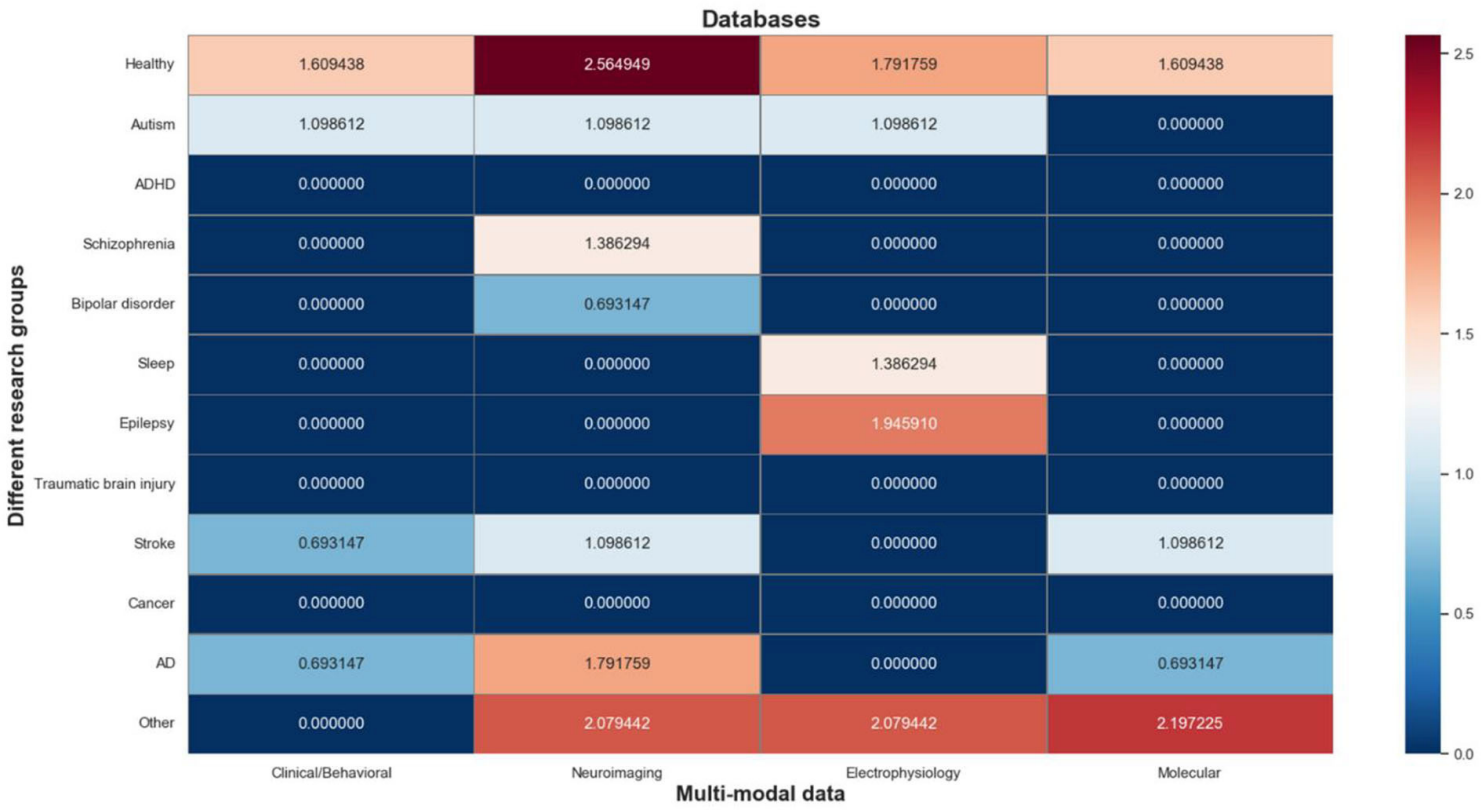
Heat map of multi-modal databases.

The multi-modal databases contain a total of 83 information objects, which are mapped to a matrix composed of research groups and multi-modal data types. As shown in Figure 6, we use a log-normalized heat-map to represent the number of informative objects distributed in each cell, where red indicates a high number of informative objects in that cell, and blue indicates a low number of that. It's worth noting that the research groups involved in these databases can be roughly divided into 4 categories: normal people (“Healthy”), mental illness (“Autism”, “ADHD”, “Schizophrenia”, “Bipolar disorder”, “Sleep”, and “Epilepsy”), organic disease (“Traumatic brain injury”, “Stroke”, “Cancer”, “AD”), and other.

**Figure 7:**
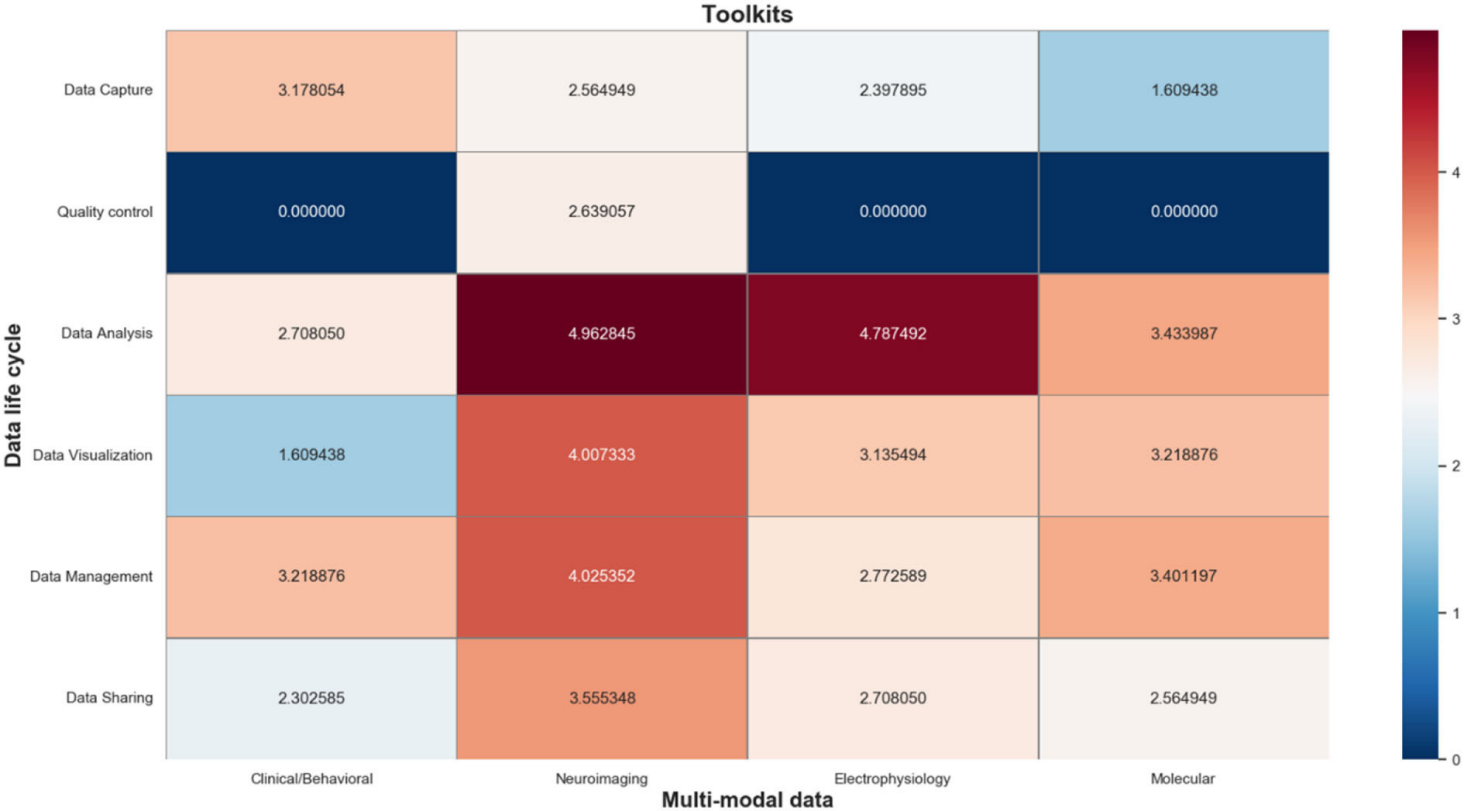
Heat map of information toolkits.

The information toolkits contain a total of 484 information objects, which are mapped to a matrix composed of different phases of the data life cycle and multi-modal data types. As shown in Figure 7, we also use a log-normalized heat-map to represent the number of informative objects distributed in each cell, where red indicates a high number of informative objects in that cell, and blue indicates a low number of that. In particular, the information objects in the data capture phase mainly include tools for multi-modal data acquisition; the information objects in the data QC phase mainly include tools for multi-modal data quality evaluation; information objects in the data analysis phase mainly include tools for simulation analysis, format conversion, data annotation, and data modeling of multi-modal data signals; the information objects in the data management phase mainly include tools that support integration and storage of multi-modal data; and the information objects in the data sharing phase mainly include tools that support anonymization, citation, and sharing of multi-modal data.

In addition, the projects/organizations contain 110 information objects, and 43 connections are established with the information objects in the multi-modal database and the information toolkits through the project attribute in the information object dictionary.

In summary, it can be seen that a large number of multi-modal databases and toolkits have been derived in different data life cycles of cohort studies, and a complete cohort study community has been gradually constructed. Next, based on the content of the constructed knowledge base, we will discuss some major research advances and underlying trends in different stages of the data life cycle.

## Multi-modal data capture

As the first step of the data flow, the data capture process pays much attention to data quality assurance. From the data validity in a single modal to the collaboration of multi-modalities, the emerging electronic data capture (EDC) software upgrade itself to adapt to both common and special occasions and environments.

### Data validity in EDC

Data verification is a verification operation to ensure data integrity and validity. When the scale of data collection changes from a single modal to multi-modal signals, at the source of data generation, data verification has always been established as the first line of protection.

The labor-intensive process of transcription from paper records to electronic records results in delay and random errors in large-scale research (Babre and Deven, 2011). Thus, the EDC becomes prevalent, but the misspelling and illegal input remain here. To solve this problem, the electronic data capture systems, such as Redcap (Harris et al., 2009), CARAT (Turner et al., 2011), CIGAL (Voyvodic, 2011), and OpenClinia ^1^, incorporate the data verification functions to check the specific logic problems and symbolic problems, in order to ensure the integrity and validity of the collected data.

Not only in the field of clinical/behavioral/electrophysiological data collection, but also in multi modalities data capturing, the data verification function plays an essential role in multi-modal EDC, such as ACQ4 provides an event detection module to monitor the collection of multi-modal data, and other examples in electrophysiology, photo-stimulation, and imaging (Luke et al., 2014). Like ACQ4, Epus is useful for meeting the needs of researchers to capture electrophysiology and photo-stimulation together (Benjamin, 2010).

Although the existing EDC systems present a data verification mechanism to ensure multi-modal data integrity and validity, areas for future development include support for a wider range of acquisition devices, and support for allowing data link to the high-throughput analysis workflow modules, with consistent data capture and provenance information, to extend the functionality of the EDC system.

### Time alignment of multi-modal data

Time alignment refers to aligning different modal data signals on the same time axis. It helps researchers not only to reveal the statistical relationship between two or more modal data signals in large-scale data sets but also to purify single modal data signals with the auxiliary of other modalities.

Platforms providing association mining across multiple modalities bind different modal data to achieve novel mechanisms or patterns in neuroscience. Brainliner, one of such platforms, provides time-aligned data signals across neurophysiological and behavioral data for assisting data-driven neuroscience and neural decoding. For example, visual images can be reconstructed and decoded from brain functional magnetic resonance imaging (fMRI) data (Emi et al., 2018).

Since the multi-modal data signals interact with each other, the collected data signals are not simply induced by experimentally designed cognitive tasks (Chang et al., 2009; Glover et al., 2015). As a result, the data signals contain extra noise, which affects the accuracy of the experimental results. For example, fMRI signals could be affected by physiological signals such as breathing and heart rate during the experiment. Therefore, the CIGAL software purifies the fMRI signals with the auxiliary of electrophysiological data including the heart rate (Voyvodic, 2011).

The benefits of time-aligned multi-modal data are obvious. However, because wearing a heart rate collection device on the tip of the left finger will cause inconvenience to keyboard operations, there is an uncertain delay deviation in the real behavior signal record. Therefore, paying attention to the convenience of experimental operation can further ensure the authenticity of the time alignment results of different data signals.

### Offline mode and local feedback of EDC

Due to the long-term and large-scale temporal-spatial distributed characteristic of the multi-sites cohort study, EDC software should meet the needs of use in special occasions or environments with limited internet access, such as remote rural areas, prisons, and medical centers. In such occasions or environments, local caching and local QC become a solution to solve offline data collection and transmission.

In order to solve the aforementioned challenges, there are currently two techniques. First, the offline mode is equipped within EDC to achieve offline caching capabilities of data. Most prevalent EDC software, such as REDCap Mobile (Borlawsky et al., 2011) and CARAT (Turner et al., 2011) have realized such functions. Second, the data QC program can be executed locally to obtain the data validity check results, instead of waiting for feedback from the central site, to solve the time delay problem of data quality feedback in an offline environment.

It can be seen that some main functions of the EDC system can be used without internet or network access. However, asynchronous updates may result in duplicate data or existing data in the centralized data management system, so these data can't be overwritten. In addition, the consistency of the EDC system version should be considered in the multi-site study.

## Multi-modal data quality control

Multi-modal quality control (QC) is a prerequisite for the data validity of most single or multi-site scientific research projects. Take the QC of neuroimaging data as an example, researchers performed qualitative and quantitative QC on the neuroimaging data to meet the needs of neuroscience research for repeatability measurement of large-scale and cross-sites neuroimaging data.

### Visual QC

In the process of neuroimaging scanning, due to factors such as head motion, gradient effects, and intensity inhomogeneity, many types of artifacts affected the final image quality. Using these image data containing artifacts without QC may lead to deviations in subsequent analysis and even wrong conclusions in neuroimaging studies. For example, studies have confirmed that these artifacts can cause inaccuracies in the segmentation of anatomical MRI images (Keshavan et al., 2017).

For this reason, researchers usually resort to the visualization functions provided by image analysis software to visually inspect different image modalities. For sMRI volumes, FSLView allows researchers to inspect neuroimaging slices in the axial, sagittal, and coronal planes (Jenkinson et al., 2012). For fMRI volumes, MRICron^2^ supports switching options for fMRI time series and offers some brain slices for visual inspection. For DTI volumes, in addition, to providing the FA, MD, and ADC images, LONI Viewer also provides the magnetic field gradient direction table for researchers to proofreading these images (Kim et al., 2019). There have also been efforts made for the quality assurance of the preprocessed neuroimaging data, such as fiber tractography extracted from DTI data (Sommer et al., 2017) and brain registration in fMRI studies (Benhajali et al., 2020).

It is not difficult to imagine that in the visual inspection of large-scale images, factors such as the professional level, fatigue degree and participation motivation of image quality raters are usually difficult to be fully and effectively controlled, thus increasing the risk of inconsistency in QC results across raters. Although researchers can avoid these effects to some extent by aggregating multiple ratings from a large pool of raters (Benhajali et al., 2020), the root cause is the lack of a standard and validated protocol to perform visual QC. Therefore, the development of standardized protocols for visual QC will produce QC ratings of higher quality on large amounts of data, which will in turn help to train machine learning models to perform automated QC, thereby reducing the burden of visual inspection.

### Automated QC and comparison to visual inspection

Some studies such as IMAGEN (Schumann et al., 2010), HCP (Marcus et al., 2011) or ABCD (Caseya et al., 2018) have obtained huge MRI datasets, in order to meet the demand for data volume in the era of big data analysis. It is very time-consuming and tedious when using visual inspection for QC of these massive datasets. Therefore, researchers have tried to use automated QC to substitute the manual QC procedure. The automated QC quantifies the image QC metrics and automatically flags images of poor quality by setting their cutoff values.

At present, some automated QC systems have been developed for checking the QC of different image modalities. For example, Oguz et al. developed the DTIPrep tool to perform QC on DTI images (Liu et al., 2014); Pizarro et al. (2016) proposed several QC metrics to describe the artifacts of sMRI images and trained a classifier based on these metrics to evaluate the quality of sMRI images. These tools usually execute QC procedures of specific image modalities on personal computers or small-size computing clusters. As a result, the use of these tools in large-scale, multi-modal image data QC work is limited. To this end, researchers have calculated a comprehensive set of standard QC metrics that have been described in the literature and developed a web-based LONI Pipeline QC system for sMRI, fMRI, and DTI (Kim et al., 2019).

However, the results from automated QC do not always fully agree with the visual inspection results (Pizarro et al., 2016; Esteban et al., 2017). There are two possible reasons for this phenomenon. First, the deterioration in image quality is caused by multiple types of noise, and the single QC metric may be used to detect one type of image artifact. In contrast, the visual assessment is often a comprehensive assessment. Second, the setting of thresholds along with the number of simultaneously occurring “bad” QC metrics may affect the consistency of the final classification results.

The development of quantitative QC metrics is critical in solving the subjectivity in visual assessment and is helpful for the development of automated QC systems for neuroimaging data. Thus, the methods of QC assessment can be replicated across multi-site datasets. However, due to the difference in the image sequence and weighting method, as well as the different degrees of motion artifacts in children and adults, the optimal cutoff values for auto QC may be allowed to be flexible scaling by the user. In addition, compared to univariate analysis that only relies on QC metrics separately, a machine learning method using multivariate modeling of QC metrics distribution may improve the accuracy of image quality classification (Pizarro et al., 2016; Fonov et al., 2022).

## Multi-modal data mining analysis

Brain network analysis has been widely considered an important method to understand the pathophysiological mechanism of many neurodegenerative diseases and mental diseases, including cognitive impairment (Chen et al., 2017; Javaria et al., 2018; Xia-An et al., 2018), Parkinson's disease (Schumacher et al., 2019) major depression disorder (Liao et al., 2018), and autistic spectrum disorder (Yu et al., 2020). Sometimes it behooves us to decide whether conclusions are obtained through a rigorous data analysis process. In making the data analysis process transparent, the development of workflow technology has increasingly satisfied our pursuit of scientific repeatability in neuroscience research.

### Multi-level analysis of brain connectivity

Human perception, cognition, and action are supported by a sophisticated, interconnected network of brain structures and functions. Thus, a number of studies analyzed brain connectivity at the macroscopic or microscopic scales, providing an important foundation for revealing the neurophysiological mechanism behind normal brain function and disease-related dysfunction. At the macroscopic scale, sophisticated neuroimaging techniques have opened up new possibilities to infer the structural and functional connectivity of brain regions. For example, Anastasia et al. proposed an automatic probabilistic reconstruction of white matter pathways based on DTI and demonstrated automatic tractography analysis in schizophrenia patients and healthy subjects (Anastasia, 2011). At the microscopic scale, Markus et al. show three-dimensional polarized light imaging (3D-PLI) can generate fiber orientation vectors of the human brain, which can be used as the basis for high-resolution fiber tract reconstruction in the human brain (Markus et al., 2011).

Recent advances in multi-scale data acquisition methods have made it easier to collect data for studying human structural and functional connectivity networks. However, since these connectivity data usually rely on indirect connectivity measures, such as DTI and fMRI, researchers need robust statistical methods to verify the validity of these connectivity data (Leergaard et al., 2012). For example, researchers have used causal reasoning algorithms to obtain effective brain connectivity information from fMRI data (Daniel and Stefano, 2014; Martin et al., 2014). Based on these effective connections, a large number of network analysis methods have been proposed to reveal complex spatiotemporal dynamics of the human developing brain. For example, by comparing the changes in the network architecture of the same brain at different spatial resolutions, Echtermeyer et al. (2011) clarified that the spatial scale and resolution play an important role in drawing conclusions based on network analysis. Similarly, He et al. (2018) proposed a developmental meta-network decomposition (DMD) approach to decompose the developmental networks into a set of temporally smooth developmental meta-networks (DMs), which may reveal the underlying changes in connectivity over brain development.

Obviously, mapping multi-scale brain connectivity analysis is the basis for comprehending the brain's complex function. Despite the numerous brain connectivity studies, we still know little about neuroanatomy and functional connectivity remains limited. In this case, researchers propose using workflow technologies to standardize the process of brain connectivity data collection and analysis. The technology will help researchers to effectively compare and combine these brain connectivity data of previous studies. These data will provide a solid foundation for the long-standing goals of achieving complete connectome maps for the human brain in the neuroscience community.

### Data processing workflow for neuroimaging

Scientific workflows are normally visualized as a collection of modules with pipes to represent the data flow from the output ports of one module to the input ports of another. With neuroscience datasets continually expanding in size, scope, and complexity, a large number of efficient processing tools need to be developed to mine more useful information from these datasets. Workflow technologies can link these tools into high-throughput processing pipelines, in order to provide the means for wide dissemination and validation of research protocols and scientific findings.

Taking neuroimaging data processing as an example, some sophisticated neuroimaging processing tools (e.g., AFNI (Cox, 1996), FSL (Jenkinson et al., 2012), ANTs (Avants et al., 2008) ^3^, SPM ^4^, FreeSurfer (Fischl, 2012), and Nipy (Millman and Brett, 2007) ^5^) have been designed to analyze multimodal imaging data. However, these tools are accessed and interfaced with in different ways, such as shell scripting (AFNI, FSL, ANTs, FreeSurfer), MATLAB (SPM), and Python (Nipy). Thus, there is no unified way to use or execute these tools in the existing pipelines. For example, SPM, written in MATLAB, does not provide a command line interface. This has resulted in the LONI pipeline (Ivo, 2009) can't interact with SPM. In this case, researchers have proposed Nipype, an open source, python-based open source software that easily interfaces with existing tools for efficiently processing of neuroimaging data (Krzysztof et al., 2011). Based on Nipype, several pipelines have been proposed for specific research purposes, such as MRIQC used for the QC of sMRI and fMRI data (Esteban et al., 2017), and Pypes used for pre-processing Positron Emission Tomography (PET), sMRI, fMRI, and DTI data (Savio et al., 2017).

Methodological improvements in the neuroimaging pipeline, such as non-linear spatial normalization and Bayesian Markov Chain Monte Carlo approaches, can dramatically increase the computational burden. Neuroimaging tools benefit from the growing number of parallel hardware configurations (multi-core, clusters, clouds, and supercomputers), and thus help facilitate data processing workflow for solving specific research problems (e.g., image registration, image segmentation, and statistical analyses). For instance, researchers have proposed BROCCOLI for parallel analysis of fMRI data on many-core CPUs and GPUs (Anders et al., 2014). Similarly, researchers have proposed ATPP ^6^ to realize the framework of brain parcellation with massive parallel computing. ATPP implements parallel computing across and within machines by means of SGE and MATLAB PCT, respectively.

Workflow technologies address the need for transparency, efficiency, and repeatability in cohort studies by providing valid and complete process records. Meanwhile, workflow technologies also provide an important opportunity to compare and combine results from previous studies *via* meta-analytic and data mining approaches. Thus, as the diversity of research applications increases, workflow technologies must be flexible for diverse research applications while being able to include new applications without modification, in order to reduce the learning curve for researchers to leverage and improve these workflows.

## Multi-modal data visualization

The brain is such an extremely complex organ, requiring researchers to interpret it from multiple levels. Benefiting from the development of multi-scale measurement methods, more and more data mining results are presented. Visualization provides an important way for researchers to gain new insights into extracting, disseminating, and interpreting these data mining results.

### Multi-scale data interactive visualization

The purpose of scientific visualization is to represent multi-modal data graphically and to facilitate the extraction and interpretation of useful information from multi-modal data by leveraging humans' abilities for pattern recognition, and intuition. To make the most of these capabilities, researchers resort to interactive visualization tools, in order to assist the analysis process of multi-scale data.

To date, a large number of interactive visualization tools have been developed to assist researchers in multi-modal data visualization. These tools focus on data visualization at the micro scale neuronal circuits and at the meso/macro scale brain regions. At the micro scale neuronal circuits, Visimpl supports researchers in visually analyzing complex neuron-level detailed brain simulations (Galindo et al., 2016). Relevant works include ShuTu (Jin et al., 2019) and VIOLA (Senk et al., 2018). At the meso/macro scale brain regions, visualization tools fall into two categories. The first is the visualization tool for a single mode, such as EEGVIS (Robbins, 2012), BrainBroswer (Tarek et al., 2014), Procortex (Gao et al., 2015), Fiberweb (Louis-Philippe et al., 2017), and webTaDat (Li et al., 2021); the second category is the visualization tools compatible with multi-modal data, such as DataView3D (Gouws et al., 2009), the virtual brain (Marmaduke et al., 2014), iBrainEEG (Rojas et al., 2016), and Visbrain (Combrisson et al., 2019).

Although much effort has been devoted to providing visualization tools compatible with multi-modal data, several areas for future development include making these tools fully compatible with Jupyter to embed the visual function into notebooks and iPython for the interactive shell, and the development of automated algorithms for automatic annotation and tracking of multi-modal data, in order to improve the efficiency of data visualization analysis by researchers.

### Visual representation of data mining results

Visualization of the data mining results may help researchers to understand their data as well as in the dissemination and exchange of knowledge. In neuroscience, the neural network model and brain atlas are important products of data mining results.

When the data mining result is applied to the neural network model, it usually appears in the publication in the form of technical illustrations supplemented with text descriptions. The description of the neural network model mainly includes the network structure, connectivity, and neuron and synapse types. With the increasing complexity of network and the demand of researchers for spatial structure information representing network connections, the traditional geometric box and arrow diagrams can no longer convey the author's true intentions clear. For this reason, in addition to using box and arrow diagrams to provide network structure information, researchers have proposed the Connectivity Pattern Tables (CPTs), which are generated by ConnPlotter to represent the spatial connection information of the network (Nordlie and Plesser, 2010). In addition, considering neuroscience is an interdisciplinary field, Neural Schematics was proposed as a unified formal graphical representation method for neural network structure, in order to further eliminate obstacles when researchers from different domains communicate neural network ideas and concepts (Matthias and René, 2013).

When the data mining result is the brain atlas, some atlas viewing tools are developed for specific atlases, for example, the BrainExplorer for the Allen Brain Map (Sunkin et al., 2012). However, the close integration of the atlas viewer and the specific atlas limits its interoperability with other atlas resources. Therefore, to decouple the atlas viewer from the specific atlas, there have been some efforts to provide standardized data exchange formats and visual viewing tools for all publicly available brain atlas, such as the Human Atlas Working Group (HAWG) data format allows atlas sharing viewing tools, data editors, and other atlas creation software. Based on this data format, researchers presented the Open Anatomy Browser (OABrower), an experimental anatomy atlas viewer for atlas interoperability (Michael et al., 2017).

Although the researchers' visualization work on complex neural network models and brain atlases validated the usefulness of Neural Schematics and HAWG concepts, respectively. However, it is worth noting that models and modeling concepts are constantly changing. Thus, the concepts built around them should be constantly changing with the need of different application domains, in order to ensure these concepts are universally applicable.

## Multi-modal data management

In neuroscience research, more and more multi-scale data are collected and archived for different research topics. Researchers have designed a large number of data management systems, in order to support the storage and retrieval of these data. The data management system needs to standardize data formats and resource description schemes for heterogeneous data, in order to facilitate the knowledge representation and integration of neuroscience.

### Some data repositories and data management systems

With the increase in the scale of research projects, some research laboratory-level data management systems are facing challenges from new technologies (e.g., data scale, QC, and complex data analysis) and society (e.g., system maintenance staff turnover and data sharing needs) (Buckow et al., 2016). Therefore, instead of reinvesting manpower to develop new software, a more practical method is to use existing solutions. These solutions can realize the electronic collection and management of neurophysiological data, and automatically upload data to the central repository for archiving.

The central data repository promotes the availability of neurophysiological data and is one of the important guarantees for reproducible research (Gorgolewski et al., 2015). The central data repository can be divided into three main categories. The first category is the original database for special populations, such as ADNI (Mueller et al., 2005; Jack et al., 2010), ABIDE (Martino et al.), NDAR (Dan et al., 2012), and ADHD-200 (Fair et al., 2013). The second category is modality-specific repositories, such as OpenfMRI ^7^ (Poldrack et al., 2013), NITRC ^8^, NeuroVult (Gorgolewski et al., 2015). The third category is derived repositories with highly processed data, such as SumsDB ^9^ (Dickson et al., 2001), BrainMap ^10^ (Laird et al., 2005), Neurosynth ^11^ (Yarkoni et al., 2011).

For multi-modal data storage and management purposes, some data management systems are designed. The existing data management systems can be mainly divided into two categories. The first category is the research project management system based on full data hostings, such as COINS ^12^ (Adam et al., 2011), NiDB (Book et al., 2013), LORIS ^13^ (Samir et al., 2011), XANT ^14^ (Marcus et al., 2007), Redcap (Harris et al., 2009), LabIS (Dimiter, 2011) and HiveDB (J-Sebastian et al., 2013). The second category is the lightweight data management system, such as odML (Lyuba et al., 2016), Expipe (Lepperd et al., 2020), Clowdr (Kiar et al., 2019), and NeuroManger (David et al., 2015).

Due to the division of the above-mentioned databases may overlap in particular research areas, the data management system further needs to support cross-database joint queries. Take AD data retrieval as an example, in addition to ADNI repository specifically for the AD population, OPENFMRI may also include FMRI resources for the purpose of AD diagnosis. In addition, different data management systems have their own independent characteristics. For example, both Redcap and XNAT systems can provide an API for automating data management tasks, LORIS and NiDB can be installed and managed locally in personal laboratories. Thus, researchers need to carefully evaluate the research conditions and requirements when choosing proper data management systems.

### Manage metadata and experimental data

Experimental neuroscience collects data with a wide range of techniques including clinical/behavioral tasks, imaging, electrophysiology, and genetics. These data cover multiple spatial and temporal dimensions. Thus, in order to meet researchers' management needs for standardized data structures, the data management process needs to deal with a wide range of metadata and experimental data formats generated by different experimental paradigms.

In neuroscience, the experimental data generated various data formats with different vendor software. For example, the formats for clinical and behavioral data are CSV, XLSX, and TXT. For imaging data, the common data format standardization includes ANALYZE 7.5, DICOM, NIFIT, GIFTI, ECAT, GE, MGH, HRRT Interfile (Cradduck et al., 1989), NRRD, Interfile, and MINC (Vincent et al., 2016). For electrophysiological data, the common data format standardization includes Opne Ephys (Adrian et al., 2014), NIX (Adrian et al., 2014). For biological samples, the common data format standard includes BioSig (Vidaurre et al., 2011), Neo ^15^, EDF+ (Kemp and Olivan, 2003), NeuroShare^16^, SignalML^17^ (Durka and Ircha, 2004), and Pandora^18^.

Metadata, which refers to the structure of data, describes other data. It can be extracted from experimental data and used as an index to retrieve experimental data. For example, an image may include metadata that describes the picture size, the color depth, the image resolution, and when the image was created. The information is self-evident for subsequent image analysis. However, metadata is rarely provided in a unified structured, comprehensive, and machine-readable form, which makes it difficult to retrieve across multiple datasets. In order to solve the above problems, researchers proposed an “open metaData Markup Language” (odML) based on extended key-value pairs (Jan et al., 2011; Lyuba et al., 2016). It uses odMLtables, which are normally represented in tabular, to organize and store complex metadata in a hierarchical structure (Sprenger et al., 2019). Similar to odML, the Neurodata Without Borders (NWB) format is defined for storing neurophysiological data and its related metadata (Jeek et al., 2020).

Furthermore, in order to better integrate and hierarchically manage metadata and experimental data in neuroscience, some open data storage specifications have been continuously proposed. These specifications include the Brain Imaging Data Structure (BIDS) for neuroimaging (Gorgolewski et al., 2016), and Hierarchical Data Format (HDF5) format (Teeters et al., 2015) as well as Exdir for the general field (Svenn-Arne et al., 2018).

In consideration of the latest use of multi-modal data, metadata acts as the indexing role. Due to the flexibility of the key-value representation of metadata, researchers could add experiment-related information arbitrarily, thus making metadata lose its meaning in sharing information across multiple datasets. Therefore, metadata inspection, through which researchers can check whether all mandatory fields exist in the data file and verify the consistency of the information in these fields, should be seriously considered and needs further discussion.

### Knowledge representation and integration in neuroinformatics

Researchers with specific research questions usually need to read up on the subject to retrieve relevant information. This retrieval process is undoubtedly time-consuming. Therefore, researchers propose a knowledge base management system for answering neuroscience questions, which can quickly help to answer research questions, thereby expediting the exposure of the still controversial or missing parts of neuroscience.

Neuroscience research has produced a lot of resources including tools, protocols, and data, to expound on the mechanism of different neuroscience phenomena. However, these resources are scattered and difficult to integrate (Bono and Hunter, 2012). A key cause of this situation is the lack of a unified semantic framework in neuroscience, which refers to unifying naming rules and granularity of resource annotations in specific fields (Gardner et al., 2008). Without the framework, the terms in the neuroscience field are full of synonyms, partial correspondences, and even homophones, making otherwise effective scientific communication unnecessarily difficult. Take neuroanatomy as an example, based on BAMS Neuroanatomical Ontology (Bota and Swanson, 2010), researchers use the projection translation method to achieve the unified correspondence of terms across different nomenclatures.

Similar works include NeuroLex.org, a semantic wiki-based website as well as a knowledge management system in the neuroscience field. It brings neurobiological knowledge into a framework, in order to allow neuroscientists to review the concepts of neuroscience, and then link thisknowledge to data sources and descriptions of important concepts in neuroscience (Larson and Martone, 2013). Another related work, ApiNATOMY (Kokash and de Bono, 2021), as a topological and semantic assembly framework, can help physiologists to capture the process interactions between neuroanatomical entities in multi-scale physiological route modeling, such as the Nephron engages in multiple coalescences with Blood Vessel leaf-distal lyphs.

Therefore, building a unified semantic framework can help to create a machine-processable multi-scale neuroscience knowledge base. Possible future expansion directions include the development of graphical tools and automated algorithms to detect the novel topological relationships between neuroscience terms in the knowledge base, in order to accelerate the construction process of the neuroscience knowledge base.

## Multi-modal data sharing

Data sharing plays an essential role in open scientific research and contributes to the reproducibility of the research, the cost performance of the funding, and the small effect identification. By sharing the research data, the low quality data features, such as missing value and noise, could be uncovered with multiple datasets comparison, thus making it possible to verify the reproducibility. Due to the costly process of data collection, sharing what we have obtained could increase the cost-benefit ratio of the funding, which obviates the need for repetitive data collection for the same research goal. Moreover, small effects could be easy to be identified by combining the shared data into large databases.

Although the benefits of data sharing are obvious, the challenges of preventing researchers to share data are self-evident, which are the concern over ethical and privacy issues, the non-standardized data sharing schema, and the low level of motivation to share from the authors. Specifically, data owners first worry about whether the content of shared data meets the ethical and legal requirements for data privacy and security (Poline et al., 2012; Poldrack et al., 2013; Gorgolewski et al., 2015). Second, they may find it difficult to integrate the shared data due to the metadata management of heterogeneous data is complex and standards are not unified (Garcia and Fourcaud-Trocme, 2009; Poline et al., 2012; Poldrack et al., 2013; Christian et al., 2014; Vaccarino et al., 2018). Moreover, the lack of widely accepted quantitative methods to highlight the contribution of shared data also restricts the motivation of data owners to participate in data sharing (Poline et al., 2012; Poldrack et al., 2013; Honor et al., 2016).

In order to overcome the aforementioned challenges, researchers and organizations have done abundant work in data security and privacy, sharing standards and schema as well as highlighting the contribution of the data owner.

### Data security and privacy

Data is usually collected in the context of solving specific scientific research problems. However, due to some public data privacy violations, the subjects' privacy is under attack. For example, researchers can combine DNA sequences with publicly available, recreational genealogy databases to re-identify subjects (Gymrek et al., 2013), which makes subjects worried about their identifiable health information being shared with unknown parties and used for an unauthorized purpose, such as advertising research or insurance (Wardlaw et al., 2011). Therefore, under the premise of complying with the ethical requirements of data privacy, some emerging technical means should also be adopted to strengthen the protection of data security.

Obtain an informed consent document from the subject is the premise of data sharing, which is the legal requirement of two international initiatives, namely the Health Information Privacy and Accountability Act (HIPAA) and the Protected Health Information (PHI). Theoretically, once we get the informed consent document from the subject, we will have the right to publish data. Practically, the Institutional/Ethical Review Boards (IRB/ERB) rarely grant researchers such right under the context of extensive data sharing in informed consent (Poline et al., 2012; Dylan et al., 2014). Therefore, to address the dilemma between data sharing and data privacy, researchers now could conduct data sharing by setting the authorized access rights of the data, such as Open Database Commons Public Domain and Dedication License (PDDL), Open Database Commons Attribution License (ODC-BY) and custom data license method (Gorgolewski et al., 2015; Makoto et al., 2016).

Although we get permission from the subject that we can publicize the data under a certain license, some data processing techniques should be applied to these data to ensure data security and privacy, such as data desensitization, data leakage prevention, and sharing highly processed derived data. To be specific, neuroimaging data should be de-identified by using customized anonymous tools (Christian et al., 2014; Vaccarino et al., 2018), such as mri_deface (Bischoff-Grethe et al., 2007), a deidentification tool for structural brain magnetic resonance images. Network and database security environments should be designed to reduce the risk of data privacy leakage, especially when allowing for querying archived data (Dylan et al., 2014). In addition, researchers have also actively advocated the sharing of highly processed derived data (Poldrack et al., 2013; Sarwate et al., 2014), such as SumsDB (Dickson et al., 2001; Van Essen et al., 2003), BrainMap (Laird et al., 2005), Neurosynth (Yarkoni et al., 2011) and BrainSpell ^19^ have shown that using differential privacy strategies uin neuroscience research is feasible (Sarwate et al., 2014; Peng et al., 2021).

Thus, in order to alleviate the concern about the privacy and security of data sharing, standards for different data modalities' sharing, for example, what content and to what extent should be anonymized as well as how to anonymize it, should be formulated. On this basis, the IRB/ERB would be able to provide guidelines for preparing ethics applications for data sharing, which could help the researchers to share data as freely as possible.

### Data sharing standards and schemes

Data collected by different equipment consist of metadata (descriptive information) and experimental data, among which there are multiple modalities, such as clinical and behavioral data, neuroimaging data, electrophysiological data, and genetic data (Vaccarino et al., 2018). However, due to the lack of a standard for data management, the metadata, and heterogeneous experimental data are organized and managed based on the privatization of different data platforms. As a result, data needs to be frequently customized and modified when integrating data, which in turn limits the communication between heterogeneous databases (Poline et al., 2012; Poldrack et al., 2013). Therefore, standardized data sharing principles, and a unified data description are urgently needed to meet the core requirement of interoperability in data integration.

Data sharing principles such as the NeuroImaging Data Model (NIDM) (Keator et al., 2013), the Cognitive Atlas Ontology (Poldrack et al., 2011), and OntoNeuroLOG (Gibaud et al., 2012), are all for special modal data annotation, have been proposed in order to win the consensus among researchers, publishers, and funders. Above all, a high-level guidelines for sharing standardized data, the FAIR Data Principles (Findability, Accessibility, Interoperability, and Reusability) have been released (Wilkinson et al., 2016), which have become the current international standard for scientific data management. Under the guidance of principles, existing studies have made progress in establishing a standard data description schema (data models, ontologies), such as XCEDE (Gadde et al., 2012) and CDISC (Souza et al., 2007).

Researchers have realized that the lack of data management standards is a hindering factor that can't be ignored in the data sharing stage, and carried out some work to establish unified data management standards. However, it is worth noting that the metadata for a specific research question still needs to be customized according to its research goals (Poldrack et al., 2013). Thus, how to obtain the balance between generalization and specifications of the schemes or to promote the nowadays models in order to be compatible with both occasions needs to be further studied.

### Highlight data sources and contributions

Even if we address the aforementioned two challenges, the lack of motivation from researchers to share data is not ignorable (Poline et al., 2012; Christian et al., 2014). It is undeniable that the research data is considered worthy of formal citation (MOONEY and Hailey, 2011), but what makes the low-level motivation is the lack of quantitative measurement of the impact of shared data which is a proxy of the contribution of the data owner. Therefore, while data sharing enhances the usability and discoverability of the scientific research community, without emphasizing the influence of shared data, it's hard to attract data owners to share data only by means of devoting themselves (Honor et al., 2016).

H-index is increasingly used as an important indicator to measure scientific research contribution and the influence of an individual. Similarly, it could be utilized to denote the influence of the dataset. For example, the ADNI's user agreement requires the ADNI consortium to be listed on all related publication's author lists, which may not meet the standards of authorship of scientific publications (Rohlfing and Poline, 2012). For this reason, some organizations have begun to develop data citation standards or guidelines, such as the Research Data Alliance (RDA) and the Joint Declaration of Data Citation Principles (JDDCP) (Starr et al., 2015). These data citation standards or guidelines aim at quantitatively measuring the impact of shared data, thus proposing a series of methods for identifying and citing data.

Researchers have investigated a variety of data identification and citation schemes, such as RRID (Bandrowski et al., 2016), Thomson Reuters PermID ^20^, PURL ^21^, Handles ^22^, and determined that the Digital Object Identifier (DOI) ^23^ is the most widely accepted and widely supported data identification and citation method. The Neuroscience Information Framework (NIF) assigns DOI to the resources and tools used in research, which are then included in publications and subsequently indexed by Google Scholar and PubMed (Gorgolewski et al., 2015).

Though the benefit of utilizing DOI in quantitatively measuring the impact of the dataset is obvious, we still need to pay attention to the existence of a single dataset appearing in multiple data repositories for avoiding the duplication of DOI. Moreover, the monitoring of improper identifiers and the standard of the landing pages of DOI should be taken into consideration as well.

## Conclusion

Faced with floods of information, such as that stored in databases targeting patients with an autism spectrum disorder or Alzheimer's disease, researchers will waste plenty of time before obtaining answers to particular questions in cohort studies. Obviously, building a neuroscience knowledge base is believed to help resolve this problem. Thus, we firstly propose a knowledge base framework that consists of projects/organizations, multi-modal databases, and toolkits related to cohort study. Then, we take the information objects about the projects/organizations, multi-modal databases, and toolkits in the Frontiers in Neuroinformatics journal as a sample input, forming the knowledge base. Meanwhile, we develop an open source complementary query tool, PDT_fninf (https://github.com/Romantic-Pumpkin/PDT_fninf), which allows interested researchers to quickly retrieve information objects from the knowledge base in question. Finally, based on the collection of information objects at different stages in the data life cycle, we analyze its research trends and draw key lessons that facilitate the discovery of new knowledge.

Although we have preliminarily constructed a knowledge base for cohort studies which has brought about the desired effect, the information objects in the knowledge base are incomplete to some extent for we merely use Frontiers in Neuroinformatics journal as input data. In our future work, therefore, we will improve the knowledge base in two ways:

Firstly, we advocate the use of the “5W-4M-6P” framework in describing different information objects in the knowledge base. Meanwhile, we hope that more researchers will transfer the framework to other journals such as NeuroImage, Neuroinformatics, Human brain mapping, etc., and contribute their findings to the open source knowledge community.

Secondly, we will employ topic mining based on natural language processing to expand knowledge base information objects. With continuous improvement, the knowledge base will provide more experience, knowledge, and innovative ideas for cohort studies, and then help yield more revealing insights based on the multi-modal databases.

## Author contributions

XL conceived the presented idea, designed the whole framework for the paper, and was in charge of overall direction and planning. HD-L collected data and developed the knowledge base query tool (PDT_fninf), conducted all the numerical experiments, and made all the figures shown in the paper. Meanwhile, HD-L and XL together wrote the manuscript and completed its revision. Both authors discussed the results, provided critical feedback, and contributed to the final draft of the manuscript.

## Funding

This work was supported by the Construction and Operation of Data Information Platform for Brain Intelligence Development (Grant No. 2021ZD0200502) and National Natural Science Foundation of China (Grant No. 62106246).

## Conflict of interest

HD-L is employed by iFLYTEK Co., LTD. The remaining author declares that the research was conducted in the absence of any commercial or financial relationships that could be construed as a potential conflict of interest.

## Publisher's note

All claims expressed in this article are solely those of the authors and do not necessarily represent those of their affiliated organizations, or those of the publisher, the editors and the reviewers. Any product that may be evaluated in this article, or claim that may be made by its manufacturer, is not guaranteed or endorsed by the publisher.

## Footnotes

^1^www.openclinica.com.

^2^http://www.sph.sc.edu/comd/rorden/mricron/.

^3^http://sourceforge.net/projects/advants/.

^4^http://www.fil.ion.ucl.ac.uk/spm/.

^5^http://nipy.org/.

^6^https://www.nitrc.org/projects/atpp.

^7^https://openfmri.org/.

^8^http://www.nitrc.org.

^9^http://sumsdb.wustl.edu/sums/.

^10^http://www.brainmap.org.

^11^http://www.neurosynth.org.

^12^http://coins.mrn.org/.

^13^https://www.nitrc.org/projects/loris/.

^14^http://www.xnat.org.

^15^http://neuralensemble.org/neo/.

^16^http://neuroshare.org/.

^17^www.signalml.org.

^18^http://userwww.service.emory.edu/~cgunay/pandora/.

^19^http://brainspell.org.

^20^https://permid.org/.

^21^http://handle.net/index.html.

^22^http://handle.net/index.html.

^23^https://www.doi.org/.
